# Iron Oxide Nanoparticles as Carriers for DOX and Magnetic Hyperthermia after Intratumoral Application into Breast Cancer in Mice: Impact and Future Perspectives

**DOI:** 10.3390/nano10061016

**Published:** 2020-05-26

**Authors:** Susann Piehler, Heidi Dähring, Julia Grandke, Julia Göring, Pierre Couleaud, Antonio Aires, Aitziber L. Cortajarena, José Courty, Alfonso Latorre, Álvaro Somoza, Ulf Teichgräber, Ingrid Hilger

**Affiliations:** 1 Institute for Diagnostic and Interventional Radiology, Jena University Hospital—Friedrich Schiller University Jena, D-07747 Jena, Germany; susann.piehler@med.uni-jena.de (S.P.); dheidi@t-online.de (H.D.); julia.grandke@gmail.com (J.G.); julia.goering@med.uni-jena.de (J.G.); ulf.teichgraeber@med.uni-jena.de (U.T.); 2 IMDEA Nanociencia & Nanobiotechnology Associated Unit (CNB-CSIC-IMDEA), 28049 Madrid, Spain; couleaud.pierre@gmail.com (P.C.); antonio.aires@imdea.org (A.A.); aitziber.lopezcortajarena@imdea.org (A.L.C.); alfonso.latorre@imdea.org (A.L.); alvaro.somoza@imdea.org (Á.S.); 3 Center for Cooperative Research in Biomaterials (CIC biomaGUNE), Parque Tecnológico de San Sebastián, 20014 Donostia-San Sebastián, Spain; 4 Ikerbasque, Basque Foundation for Science, 48013 Bilbao, Spain; 5 Laboratoire Croissance, Réparation et Régénération Tissulaire (CRRET), Université Paris EST Créteil, 94010 Créteil, France; courty@u-pec.fr

**Keywords:** magnetic hyperthermia, magnetic nanoparticles, doxorubicin, breast cancer, mouse model

## Abstract

There is still a need for improving the treatment of breast cancer with doxorubicin (DOX). In this paper, we functionalized magnetic nanoparticles (MNPs) with DOX and studied the DOX-induced antitumor effects in breast cancer cells (BT474) in the presence of magnetic hyperthermia (43 °C, 1 h). We show that i) intratumoral application of DOX-functionalized MNPs (at least at a concentration of 9.6 nmol DOX/100 mm^3^ tumor volume) combined with magnetic hyperthermia favors tumor regression in vivo, and there is evidence for an increased effect compared to magnetic hyperthermia alone or to the intratumoral application of free DOX and ii) the presence of the pseudopeptide NucAnt (N6L) on the MNP surface might well be beneficial in its function as carrier for MNP internalization into breast cancer cells in vitro, which could further augment the possibility of the induction of intracellular heating spots and cell death in the future.

## 1. Introduction

Chemotherapeutic drugs play an important role in cancer treatment. Most of them show complex mechanisms of action. One example is the drug doxorubicin (DOX), an anthracycline antibiotic, which intercalates with DNA, interrupts topoisomerase II activity and induces free radicals. These effects induce oxidative damages and DNA double-strand breaks [1,2,3,4,5] in target cells, which in turn, inhibit cell proliferation, induce cell cycle arrest and/or lead to cellular apoptosis [6,7]. 

In general, DOX is the most potent and frequently used chemotherapeutic drug for various malignancies, including breast cancer, lung cancer and several aggressive lymphomas [4,8]. However, the aforementioned cellular effects are not specific for cancer cells. Accordingly, DOX can also damage healthy tissue and cells to a considerable extent. Typical toxic side effects include myelosuppression, mucositis, stomatitis, alopecia and some severe complications, such as cardiotoxicity and hepatotoxicity [8,9,10]. In consequence, it is difficult to achieve a highly effective DOX dosage particularly in solid tumors after its systemic application to the body, and this fact distinctly limits its utilization in the clinical environment.

To overcome this problem, there have been several approaches already proposed with the aim to reduce the systemic toxicity of DOX, while retaining the drug delivery and efficacy to target tissue or improve it at best. One strategy is the use of specific nanotherapeutic delivery systems, which provide the possibility to selectively accumulate DOX at the target tissue [11]. For example, DOX-functionalized magnetic nanoparticles (MNPs) were studied for their ability to release the drug via magnetic means [12], or via their features to react in presence of pH changes [13]. 

Another promising approach consists in the use of magnetic iron oxide nanoparticles (MNPs) in its double function as a carrier for DOX and inductor of magnetic heating (magnetic hyperthermia). In general, MNPs administrated to organisms usually consist of a core of biocompatible iron oxides (magnetite or maghemite) and are coated with a shell of polysaccharides or other biocompatible molecules [14]. When exposing them to an alternating magnetic field (AMF) of appropriate amplitude and frequency, they release heat due to loss processes during the reorientation of the magnetization in the magnetic field, or due to frictional losses in the case of the nanoparticle being able to rotate in the surrounding medium [15]. The released heat is known to modify the normal structure of phospholipids, proteins, and nucleic acids and it transforms these cellular structures to a disordered state [15,16]. Further typical biological effects of hyperthermia include an increased fluidity of cellular membranes, inhibition of amino acid transport, impairment of certain DNA repair processes, and damage to the cytoskeletal system [16,17,18,19].

Nevertheless, some studies on DOX-carrying MNPs and magnetic hyperthermia reported up to now utilize temperatures below the critical hyperthermia threshold (43 °C for 60 min) [20], and some DOX- carrying MNP formulations were synthesized for utilization in magnetic hyperthermia but without comprehensive pathobiological examinations [21].

In the present study, we analyze the effects and perspectives of antitumor effects in breast cancer cells, when DOX is functionalized to MNPs, applied intratumorally and used in combination with magnetic hyperthermia. In this context, we investigate if: (i) DOX covalently attached to MNPs in combination with hyperthermia favor tumor regression in vivo. (ii) Further on to elucidate further beneficial treatment effects by utilization of triggered carrier internalization into breast cancer cells, the presence of the multivalent pseudopeptide NuCant (N6L) [22] on the surface of DOX-functionalized MNPs in combination with magnetic hyperthermia was studied in vitro. Namely, nucleolin and/or nucleophosmin [22,23] were shown to be present on the surface of different human cancers [24]. After binding, cell surface nucleolin is able to shuttle N6L (at least non-MNP functionalized) inside the cell, even to the nucleus, where N6L mediates several anticancer effects (e.g., cell growth inhibition [25] and cell death [26]). Nevertheless, it is still not known, if N6L could potentiate the internalization of DOX-functionalized MNPs into cancer cells.

## 2. Materials and Methods 

### 2.1. Synthesis of Magnetic Nanoparticles (MNPs)

Superparamagnetic iron oxide nanoparticles (MNPs), named MF66, were synthesized by co-precipitation technique as described previously [27]. They were coated with a dimercaptosuccinic acid (DMSA) and covalently functionalized either with the chemotherapeutic drug doxorubicin (DOX; MF66-DOX; 40 µmol DOX/g iron) for in vivo studies or with the pseudopeptide NuCant (N6L; MF66-N6L; 3.5 µmol N6L/g iron), and lastly with both (MF66-DOX-N6L; 40 µmol DOX/g iron; 3.5 µmol N6L/g iron) for in vitro analysis as described previously [28]. The amount of immobilized N6L, DOX or both on the MF66-MNPs was quantified as described previously [28].

### 2.2. Characterization of MF66-MNPs

The core size of the non-functionalized and functionalized MNPs, the hydrodynamic diameter (expressed as Z-average size) and the Zeta potential (PBS and water, pH 7.4) of the MNPs were determined using Zetasizer equipment (Zetasizer Nano ZS, Malvern Instruments, Herrenberg, Germany). The morphology of the MNPs and TEM images of uncoupled MF66 nanoparticles were described previously [29]. To assess the heating potential of the different MF66 formulations, the specific absorption rate (SAR) was determined in different environments (ferrofluid, immobilized in 10% polyvinyl alcohol and immobilized in cells 24 h after incubation with MNPs at a concentration of 100 µg iron/mL) [29]. In order to compare SAR values with data in the literature, the intrinsic loss power (ILP, i.e., the SAR value normalized to field strength and the frequency of the magnetic field) was calculated as described elsewhere [29].

### 2.3. Cell Culture

The BT474 cell line, a human breast invasive ductal carcinoma cell line, was selected because of their known sensitivity against magnetic hyperthermia and doxorubicin [30,31]. BT474 cells were cultivated at 37 °C in a humidified atmosphere containing 5% CO_2_ and maintained in DMEM with 10% (*v*/*v*) fetal bovine serum (FBS) supplemented with glutamax I, sodium pyruvate and glucose (4.5 g/L) (all products from Gibco^®^, Paisley, Scotland, UK). Cells were passaged after reaching up to 90% of confluence. 

### 2.4. Murine Xenograft Model, Tumor Implantation and In Vivo Magnetic Hyperthermia

All animal experiments were performed in accordance with international guidelines (we followed the rules of the Declaration of Helsinki) on the ethical use of animals and they were approved by the regional animal care committee (ethical approval code 02-068/11, approval date 21st of February 2012; Thüringer Landesamt für Verbraucherschutz, Bad Langensalza, Germany). Except for tumor implantation, all procedures were performed under inhalation anesthesia using isoflurane in O_2_ flow (2%). Animals were cared humanely during the whole experimentation period. They were maintained under artificial day-night cycles and received food and water ad libitum. At 3 days before subcutaneous BT474 cell pellet implantation, a 17 β-Estradiol pellet (0.36 mg; Innovative Research of America) was implanted subcutaneously in the region between shoulder and neck of female athymic nude mice (Hsd:Athymic Nude-Foxn1^nu^, Harlan Laboratories, NM Horst, The Netherlands) to support in vivo BT474 cell growth. Afterwards 200 µL Matrigel^®^ containing 2 × 10^6^ BT474 cells was injected subcutaneously on the rear backside of the mice. When tumors reached a volume of 200 mm^3^, animals were divided into four independent treatment groups: Animals of group 1 (therapy group) received an intratumoral injection of MF66-DOX MNPs (0.24 mg iron and 9.6 nmol DOX/100 mm^3^ tumor volume) and were exposed to an alternating magnetic field (circular coil, AMF conditions: H = 15.4 kA/m; f = 435 kHz) for 1 h. This MNP formulation was used since—based on its components—the likelihood for clinical translation was considered to be highest among all tested formulations in vitro. Group 2 was used to investigate the impact of non-functionalized MF66-MNPs on tumor growth. In this context, mice were injected intratumorally with MF66-MNPs (0.24 mg iron/100 mm^3^ tumor volume) and exposed to an alternating magnetic field at the same conditions as described above. Group 3 was used to assess the impact of DOX in the therapy (Doxorubicinhydrochloride, cell pharm GmbH, Hannover, Germany), whereby the animals received intratumorally DOX (9.6 nmol DOX/100 mm^3^ tumor volume, comparable to DOX concentration functionalized to MNPs) but no hyperthermia treatment. Group 4 was used to assess the normal tumor growth in absence of any treatment (intratumoral injection of aqua dest). Tumor surface and rectal temperatures were monitored by fiber optic temperature sensors, as described previously [27]. 

### 2.5. In Vivo Effects of Magnetic Hyperthermia on Tumor Volume, Temperature Distribution in Tumor Tissue and Blood Count 

Tumor volume was monitored every three or four days as described elsewhere [32]. The assessment of temperature distribution within the tumor area was determined as previously described [29]. Furthermore, on day 1, 14, and 28 after the initial treatment, levels of red and white blood cells, as well as the amount of hemoglobin, were determined via hematologic analyses (Sysmex pocH-100i, Sysmex GmbH, Norderstedt, Germany). 

### 2.6. MNPs Distribution In Vivo

28 days after hyperthermia treatment, all mice were sacrificed and organs (kidneys, liver, spleen, lungs and heart), muscle tissue and tumors were extracted, dried, and incinerated as described elsewhere [22]. Aliquots of each sample were measured using flame atomic absorption spectrometry (AAS) (AAS 5 FL, Analytik Jena AG, Jena, Germany).

### 2.7. Determination of Cellular Uptake of MNP Formulations

BT474 cells were incubated with the different MNP formulations for 24 h. Afterwards quantification of the intracellular iron content was performed using flame atomic absorption spectrometry (AAS) as previously described [29].

### 2.8. In Vitro Magnetic Hyperthermia

BT474 cells (e.g., 5 × 10^6^ cells) were incubated with the respective MNP formulation in a concentration of 100 µg iron/mL for 24 h at 37 °C and 5% CO_2_. In order to assess the impact of the drugs *per se* on cell viability, experiments were performed with non-functionalized (free) DOX and/or N6L at comparable concentrations as in the functionalized modality (100 µg iron/mL, 4 µmol DOX/100 mg iron and 0.35 µmol N6L/100 mg iron). Non-internalized MNPs (and free ligands) were removed by washing steps. Cells without any additives were used to represent the normal metabolic state and referred to as native cells. Afterwards cells were exposed to an alternating magnetic field (AMF conditions: H = 23.9 kA/m, f = 410 kHz) and magnetically heated to a target temperature of 43 °C for the duration of 1 h (+AMF group) or they were placed in an incubator at 37 °C for the same period of time (–AMF group). Temperature dose was monitored during AMF via fiber optic temperature sensors and thermometers. The workflow of the conducted in vitro experiments with this cell line is shown in the supplements (Figure S1).

### 2.9. Analysis of Cell Viability

Viability of BT474 cells was assessed 24 h or 48 h post hyperthermia (HT) based on the dehydrogenase (DH) activity using the alamarBlue Cell Proliferation Reagent according to the manufacturer’s instructions. Fluorescence was measured (Tecan Infinite M1000, excitation/emission: 530–560 nm/590 nm). The relative dehydrogenase activity (rDH) was determined by normalizing the measured fluorescence of the appropriate cells to the fluorescence of untreated cells. In order to assess the impact of the drugs *per se* on cell viability, experiments were performed with non-functionalized (free) DOX and/or N6L at comparable concentrations as in the functionalized modality (see above).

### 2.10. Microscopy

To investigate the influence of magnetic hyperthermia on morphological changes and detachment of BT474 cells at 24 h and 48 h after treatment, light microscopy (Evos XL imaging system, Thermo Fisher Scientific, Dreieich, Germany) was used.

### 2.11. Prussian Blue Staining for Iron Detection in Cells in Vitro

Internalization and cellular uptake of MNPs (MF66, MF66-DOX 100 µg iron/mL, respectively) and of the free agent DOX (4 nmol/mL) in BT474 cells were determined as described previously [27]. 

### 2.12. Statistics

Data was potted as mean and standard deviation of the mean. The level of significance was assessed by the utilization of the Mann–Whitney U test by comparison of treated samples or animals versus 37 °C treated samples or untreated animals. *p* values of *p* ≤ 0.05 (*), *p* ≤ 0.01 (**) and *p* ≤ 0.001 (***) were considered to be significantly different.

## 3. Results

### 3.1. MF66-MNP Formulations Show Good Heating Potential

All MNPs exhibited an iron oxide core diameter of approximately 12 nm (Table 1). In general, the conjugation of MF66 increased the hydrodynamic diameter, whereby the bi-functionalized (MF66-DOX-N6L) MNPs showed the largest increase in size (Table 1). The immobilization yields were 80% for DOX and 70% for N6L with loads of 40 μmol DOX/g iron and 3.5 μmol N6L/g iron, respectively. The zeta potential decreased upon functionalization (Table 1). The colloidal stability of the formulations was preserved under physiological conditions.

**Figure 1:**
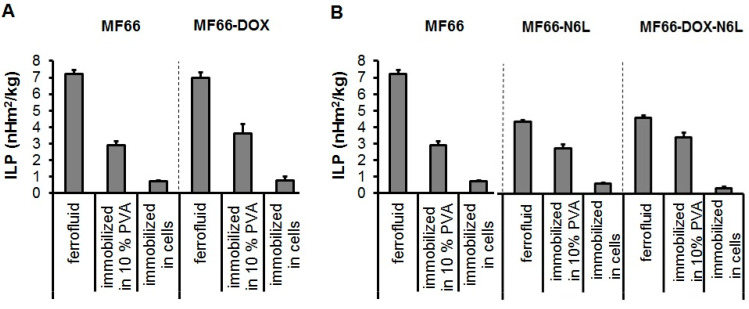
Characterization of the heating potential of the MF66-DOX (**A**) and the bi-functionalized MF66-DOX-N6L MNP formulations (**B**). MNPs were suspended in water or immobilized in 10% polyvinyl alcohol (PVA) as well as in BT474 cells. Mean and standard deviation of the mean of n = 3.

The nano-formulations were stable over one month in PBS at 4 °C and one week at 37 °C, as determined by their colloidal stability parameters (zeta potential and hydrodynamic diameter) that remained constant. The size distribution of the nano-formulations showed almost no aggregation (low percentage in terms of MNP number; Figure S2). As expected, all investigated MF66-DOX MNP formulations displayed highest ILP (intrinsic loss power) values in water suspension and an ILP reduction upon increasing the degree of immobilization (10% polyvinyl alcohol and cells; Figure 1A). 

Nevertheless, a strong ILP reduction occurred during immobilization of the bi-functionalized (MF66-DOX-N6L) MNPs in BT474 cells, but these MNPs still exhibited a good heating potential (Figure 1B). 

### 3.2. DOX-Functionalized MNPs with Magnetic Hyperthermia Cause Tumor Regression in a BT474 Xenograft Model

**Figure 2:**
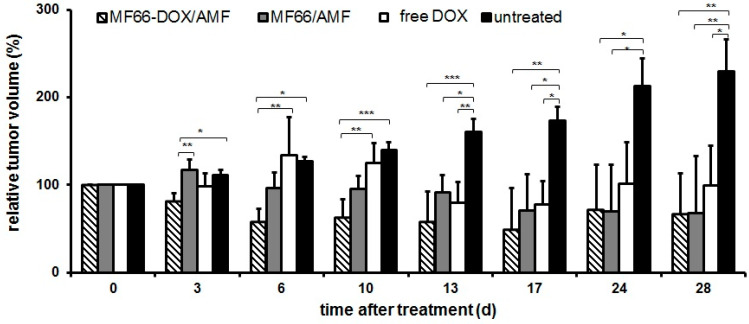
DOX-functionalized MNPs and magnetic hyperthermia significantly reduces tumor volumes in vivo. Tumor volumes (volume percentage to day 0 before treatment), mean ± standard deviation (n ≥ 4 mice per group), (*) *p* ≤ 0.05, (**) *p* ≤ 0.01 and (***) *p* ≤ 0.001.

The presence of magnetic hyperthermia combined with DOX-functionalized (MF66-DOX) or non-functionalized MF66-MNPs led to a distinct reduction of tumor volumes in mice compared to untreated ones during the period of investigation (28 days; Figure 2). Here, the relative tumor volumes decreased up to 50% compared to the initial volume measured at the beginning of the experiments (Figure 2). In the case of treating tumors with DOX-functionalized (MF66-DOX) MNPs, the tumor volume regression was obvious at six days post-treatment, whereas this effect was delayed (17 days post-treatment) when non-functionalized MF66-MNPs were applied. The additive effect of DOX and magnetic hyperthermia in reducing tumor volumes (by utilization of functionalized MNPs) was detectable up to day 17. Interestingly, the treatment of mice with the free ligand DOX (comparable concentrations as coupled to MNPs) initially seemed to promote tumor growth up to day 10. Later on, tumor growth was rather inhibited, but the effect was comparable with functionalized DOX and non-functionalized MNPs in combination with magnetic hyperthermia (no statistical difference; Figure 2). Particularly in relation to DOX-functionalized (MF66-DOX) MNPs, a high percentage of the tumor surface was covered with temperatures higher than 43 °C (Figure S3).

### 3.3. When Functionalized to MNPs, DOX Does not Exert any Transient Effect on Blood Cells and the Intratumorally Administered MNP Formulations Remained in the BT474 Tumors 

**Figure 3:**
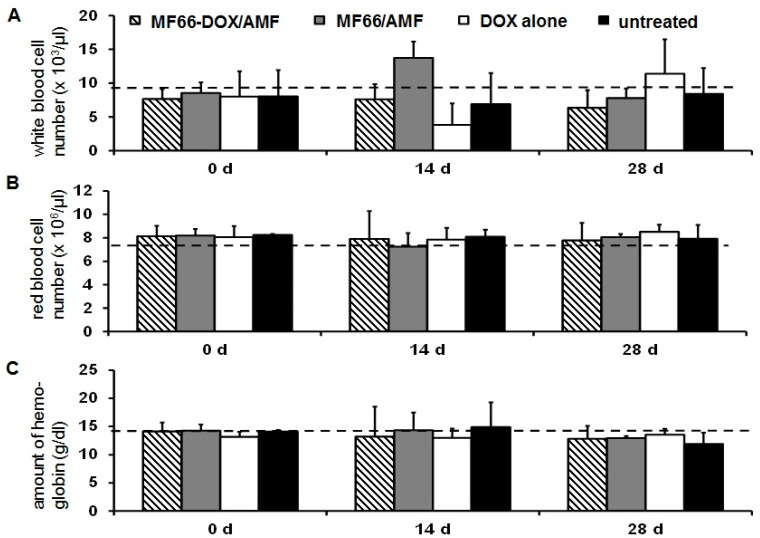
MNPs functionalized with DOX exert a good blood biocompatibility. White blood cells (**A**), red blood cells (**B**) and hemoglobin concentration (**C**). Mean ± standard deviation (n ≥ 4 mice per group). Dashed lines refer to reference values (Harlan Laboratories, Venray, The Netherlands; http://www.harlan.com).

With consideration of the effect on the blood composition of tumor-bearing mice, the presence of non-functionalized MF66-MNPs combined with magnetic hyperthermia led to a temporary increase of white blood cells (14 days after treatment, Figure 3A). By contrast, the free ligand DOX remarkably reduced the number of white blood cells (14 days after treatment, Figure 3A). Both effects reversed to normal values later on (28 days post-therapy). The number of red blood cells, as well as the hemoglobin concentration, was not altered (Figure 3B,C). 

**Figure 4:**
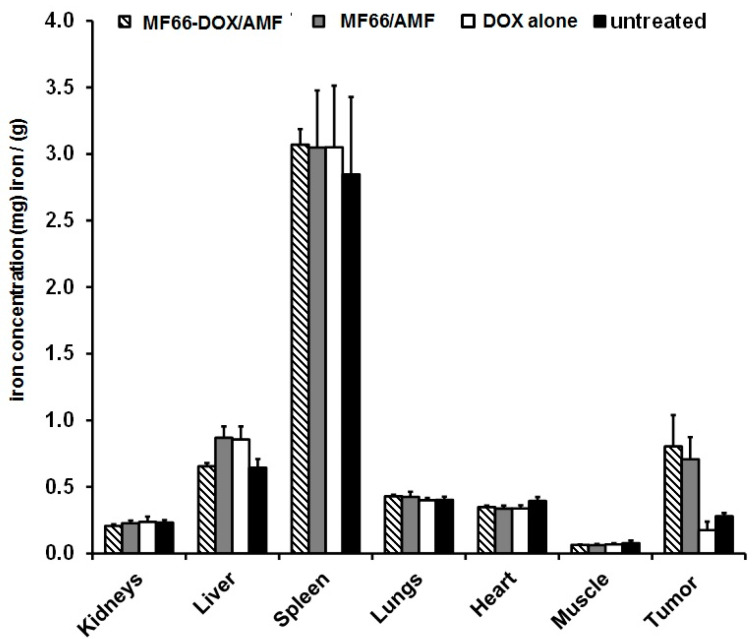
The intratumorally injected MNPs remain within the tumor after hyperthermia treatment. Iron concentration in selected organs 28 days after magnetic hyperthermia treatment. Mean and standard deviation of n ≥ 4 animals per group.

Moreover, the DOX-functionalized MNPs did not have an effect on the iron concentration in different organs in comparison untreated animals (Figure 4), when intratumorally injected. This also applies for the magnetic hyperthermia application. 

**Figure 5:**
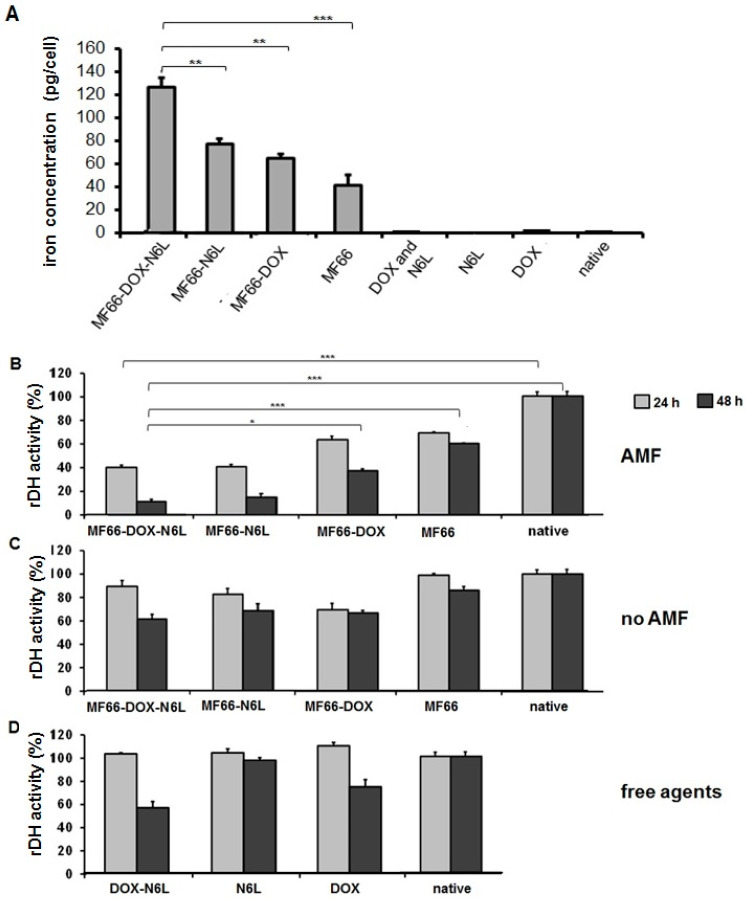
The additional ligand N6L on the surface of DOX-functionalized MNPs increases their internalization into BT474 cells and further inhibits tumor cell growth in combination with magnetic hyperthermia in vitro. (**A**) Intracellular iron content measured in the cells at 24 h after incubation with MNP formulations. Mean ± standard deviation of n ≥ 6 samples/group, *p* ≤ 0.01 (**) and *p* ≤ 0.001 (***). (**B**) The relative dehydrogenase activity was measured 24 h or 48 h after incubation with MNP formulations and the alternating magnetic field (AMF) treatment, (**C**) MNP application without AMF and (**D**) treatment with the free ligands. AMF settings: H = 23.9 kA/m, f = 410 kHz, 43 °C for 60 min. Means and standard deviation of three individual experiments with three parallels each, (*) *p* ≤ 0.05 and (***) *p* ≤ 0.001.

The additional presence of N6L on the surface of DOX-functionalized MNPs augmented their internalization into BT474 cells (Figure 5A), and in combination with magnetic hyperthermia it potentiated the MNP’s cytotoxic effect on BT474 cancer cells in vitro.

The in vitro studies related to the anti-tumor effects when DOX-functionalized MNPs were additionally coupled with the carrier N6L (MF66-DOX-N6L) for internalization into breast cancer cells (BT474) revealed the following: (a) MF66-DOX-N6L MNPs exhibited a higher cytotoxic potential (relative dehydrogenase activity) compared to the single functionalization either with N6L or DOX or the native MNPs (MF66) in the presence of magnetic hyperthermia (Figure 5B); (b) in the absence of magnetic hyperthermia, the cytotoxic potential of the MF66-DOX-N6L MNPs was only moderate, and not distinctly different from the single MNP functionalization (DOX or N6L, Figure 5C); and (c) the exposure of cells to the free agents (DOX and/or N6L) at comparable concentrations as bound to the MNPs (see above) showed that cytotoxicity was not distinctly different (absence of hyperthermia, Figure 5D).

**Figure 6:**
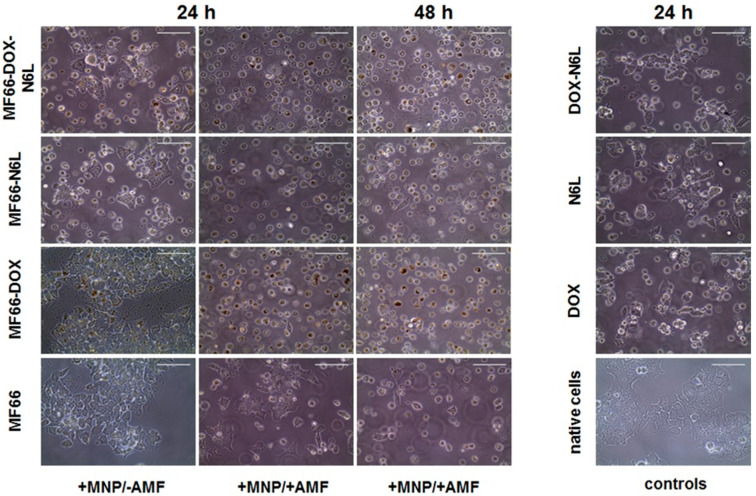
Magnetic hyperthermia in combination with ((DOX and/or N6L) MNP formulations induced morphological changes and detachment of BT474 cells. Representative microscopic images of BT474 cells, which were incubated with the different MF66 formulations (100 µg Fe/mL, 24 h); 24 h or 48 h post magnetic hyperthermia (+MNPs/+AMF), MNPs control (+MNPs/−AMF), free agent controls (DOX, N6L or both) as well as untreated controls (native BT474). Scale bar = 100 µm.

Microscopic analyses clearly revealed morphological changes and a detachment of BT474 cells after the combined treatment with magnetic hyperthermia and bi-functionalized (MF66-DOX-N6L) MNPs as well as with DOX-functionalized (MF66-DOX), such effects were most pronounced at 48 h after treatment (Figure 6). Here, the majority of cells showed rounded phenotypes (Figure 6). Moreover, fluorescence microscopy revealed the presence of DOX and iron inside the cells after exposure to the MNPs (Figure S4). 

## 4. Discussion

The results presented in this study clearly demonstrated that (i) DOX-functionalized MNPs in combination with hyperthermia favors tumor regression in vivo but did not completely eliminate them, (ii) the presence of N6L on the surface coating of DOX-functionalized MNPs increased their internalization into a breast cancer cell line and potentiated the cytotoxic potential of the anticancer drug in vitro.

In particular, in water suspension, the presence of DOX on the surface of MNPs did not lower the ILP compared to non-functionalized MNPs, but the presence of N6L and/or DOX on the surface coating of MNPs distinctly did so. This is not due to clustering of the MNPs, since PDI and zeta potential were almost similar among all formulations. The reason could be an increase in size after functionalization and therefore a reduced Brownian relaxation, which corresponds to previously described features in the literature [27]. Despite of it, the bi-functionalized MNPs still showed good heating potential. Moreover, the nano-formulations exhibited almost no nanoparticle aggregation in water (percentile of MNP number very low). For this reason, they are very well applicable for intratumoral application as used in the present study for in vivo studies in mice.

Our in vivo experiments indicated a noticeable reduction of tumor volumes, particularly after treatment with DOX-functionalized MNPs and hyperthermia. These effects were most pronounced between day 6 and 17 days after treatment. The effect of DOX and hyperthermia seemed to be additive during this time period. The additive effects [33] are the result of the combined metabolic effects of DOX, such as intercalation with DNA and causing DNA double-strand breaks, and those of hyperthermia, including changes in DNA stability, protein conformation and/or expression as well as production of reactive oxygen species [1,31,34,35].

The fact, that DOX coupled to MNPs is basically able to interact with DNA is corroborated by: (1) the knowledge that upon degradation in lysosomes DOX is released from the MNPs, and as a free molecule DOX is able to migrate to the cell nucleus [36]; (2) previous investigations showing that DOX-loaded MNPs (e.g., electrostatic coupling; in absence of hyperthermia) exert a low intrinsic cytotoxicity [27,36,37]; and (3) the tumor reducing effect of our covalently coupled DOX-MNPs (4 µmol DOX/g Fe) is lower compared to that of the electrostatically coupled ones (40 µmol DOX/g Fe, [27]). All mentioned relationships indicate that the tumor reducing effect of our DOX-functionalized MNPs was due to a synergistic mechanism between DOX and hyperthermia, even though the comparably lower antitumor effect compared to the electrostatic MNP functionalization in former studies might well be due to a reduced number of DOX molecules per mg Fe and also due to the fact that covalent conjugation attenuates the release of DOX from the MNPs to some extent.

Interestingly, the additive effect of hyperthermia and DOX-functionalized MNPs in reducing tumor volumes was detectable up to day 17 post-therapy. Later on, the effects were less obvious. In general, the combined therapy with DOX-functionalized MNPs and magnetic hyperthermia was more efficient in comparison to intratumorally applied DOX at equivalent concentrations. The fact that tumors were not completely eliminated show that more than one therapy session (including the intratumoral application of MNPs) might be necessary in order to achieve long-lasting anti-tumor impact. One important reason is that due to irregularities in the intratumoral MNP distribution, at least in the used tumor model, regions of temperature underdosage might occur in some cases. It also may be conceivable that the covalent coupling of DOX to MNPs might implicate a reduced ability of DOX to be released into intracellular space compared to an electrostatic binding of this chemotherapeutic agent. Namely, DOX (0.22–0.55 mg/ kg body weight, median temperature dosage CEM43T90 for MF66-DOX: 10 min) electrostatically coupled to MNPs led to a significant reduction of tumor volumes as already reported [27].

Interestingly, by comparing the in vivo effect of the free DOX in relation with the DOX-functionalized MNPs in combination with hyperthermia our data indicate that there is almost no difference between both. This means that the functionality of DOX per se is not distinctly impaired upon MNP functionalization. The fact that a transiently increased tumor growth was observed in vivo in the presence of free DOX compared to the other treatment groups in vivo but it was not higher than the non-treated animal group, could be attributed to hormesis effects [38] at low drug concentrations in the absence of hyperthermia. The observed transient reduction of white blood cells of the animal group, which intratumorally received free DOX, might well be attributed to an extravasation of the drug from the tumor into the blood compartment.

In order to assess the perspectives of our DOX functionalized MNPs and magnetic hyperthermia, we performed further studies on BT474 cells in vitro with utilization of the bi-functionalized nanoparticles (N6L and DOX). Interestingly, the bi-functionalized nanoparticles (N6L and DOX) are internalized in a significantly higher extent (*p* ≤ 0.01) compared to the other investigated counterparts. Possible reasons are: (1) increased diameter of the bi-functionalized MNP compared to the mono-functionalized or non-functionalized ones, (2) the particular surface features after functionalization, and (3) the formation of a protein corona on the MNP with beneficial features for cell internalization particularly when both, DOX and N6L, are attached to their surface. Moreover, the pseudopeptide N6L is known to bind to the cell surface proteins nucleolin and nucleophosmin that are overexpressed in most tumor cells [22,23,25,26]. Competition studies using the free ligand N6L, N6L-functionalized MNPs and MDA-MB231 cells were already performed showing the specificity of N6L binding [39]. Another mechanism by which N6L strengthened the internalization of MNPs includes its electrostatic interaction with sulfated glycosaminoglycans (GAGs); these are molecules associated with the cell surface [23,39]. In this context, the negatively charged sulfated GAGs can easily interact with the positively charged N6L. This fact is supported by several studies indicating glycosaminoglycan-specific roles in cancer biology [39,40]. In the case of the MNPs functionalized with both, DOX and N6L, the internalization effects of each compound are very likely to be additive, resulting in a distinctly improved cellular uptake, which in turn, is beneficial for therapeutic purposes.

The remarkable reduction of viability of BT474 cells in the presence of the bi-functionalized MNPs and magnetic hyperthermia could be due to activation of apoptotic processes. However, mechanisms other than apoptosis might have led to reduced cell viability, too [41,42,43,44,45]. Accordingly, besides apoptosis, several other mechanisms are leading to programmed cell death, including autophagic cell death, paraptosis, pyroptosis, oncosis, programmed necrosis and caspase-independent cell death [43,44]. Additionally, it is well conceivable that DOX is additionally acting through specific autophagic pathways as shown for resveratrol, a type of polyphenol antioxidant that is found in various plants and fruits [46]. Nevertheless, the reduced cell viability indicates the presence of a portion of surviving cells after treatment. This in turn, supports the induction of cellular repair processes and a proliferation of cells later on. Previous work figured out that the treatment with DOX inhibitors induces cyclin B1 accumulation, leading to G2/M phase arrest in cells in vitro [47,48]. Moreover, certain drugs can arrest cells in the G2/M phase then induce DNA repair rather than cell death [49]. Indeed, the additional presence of N6L on DOX functionalized MNPs (also see below) combined with hyperthermia increased the uptake into the BT474 cells in vitro, but seems not to further influence mechanisms investigated here. With consideration of the in vitro studies with the bi-functionalized MNPs and the single or native control MNP formulations, DOX seems to be released from the MNP in a similar manner as it has been shown in relation in a previous study [27]. The internalization of DOX via a MNP carrier into cancer cells provides the possibility of an enhanced mechanism of DOX-related actions, including oxidative damage, DNA double-strand breaks and decreased cellular growth. Basically there are two internalization mechanisms of DOX-functionalized MNPs possible: a) DOX is released from the MNPs outside the cell in the tumor environment, and b) DOX enters the cell due to endocytosis of nanoparticles and is released inside the cell into acidic intracellular vesicles, such as endosomes and lysosomes, later on [50,51]. In case that DOX is released outside the cell, the drug is able to interact with cell membranes by insertion into the lipid matrix [51,52,53]. Interestingly, within cell membranes, anionic phospholipids were shown to be important targets for this hydrophobic agent [54]. Moreover, passive diffusion is another mechanism how free DOX enters the cell [54]. This mechanism is not specific and selective for cancer cells and therefore represents a major disadvantage of DOX in the conventional mode of delivery. Furthermore, the exposure of cells with internalized MNPs to a magnetic field generates intracellular heating spots, resulting in an immediate destruction of cells [27,34,55,56]. This effect was also observed by morphological changes of the BT474 cells in this study. In absence of a magnetic field, the functionalized MNPs influence the viability of BT474 cells to a lesser extent. Based on these results, we concluded that the reduced viability of BT474 cells is due to the combination of heating and the anticancer drug DOX. Both effects potentiate the cytotoxic effects on BT474 cancer cells.

The fact that the mentioned effects on tumor cells in vitro and in vivo are due to the intended therapeutic effect and not by the intrinsic cytotoxic potential of the nanomaterials is shown in previous investigations of our group. Hereto, a good biocompatibility of the DOX and/or N6L (electrostatically) conjugated as well as the unconjugated MNPs (MF66) has been demonstrated [27,29,57]. 

To sum up, the intratumoral application of DOX-functionalized MNPs (at least at a concentration of 9.6 nmol DOX/100 mm^3^ tumor volume) combined with magnetic hyperthermia favored tumor regression in vivo, and there was some evidence for an increased effect compared to magnetic hyperthermia alone or to the intratumoral application of free DOX. The fact that tumors could not be completely eliminated in vivo might be the result of the comparatively low amount of DOX coupled per unit iron with reference to Kossatz et al. [27] as well as the limited delivery of covalently coupled DOX in comparison to electrostatic attachment to the MNPs. In the future, the presence of the pseudopeptide N6L on the MNP surface might well be beneficial in its function as a carrier for MNP internalization into breast cancer cells, which could further augment the possibility of the induction of intracellular heating spots and, in the end, tumor cell death. 

## 5. Patents

Ingrid Hilger declares that she is holding a patent DE 10 2005 062 746. Jose Courty declares that he is holding the patents WO2009141687A1 and WO2007125210A2.

## Figures and Tables

**Table 1:** Characterization of non-/functionalized MF66-magnetic nanoparticles (MNPs).

	MF66	MF66-DOX	MF66-N6L	MF66-DOX-N6L
Core size (nm)	11.7	11.7	11.7	11.7
Coating	DMSA	DMSA	DMSA	DMSA
Functionalization (per g iron)	none	40 µmol DOX	3.5 µmol N6L	40 µmol DOX 3.5 µmol N6L
Hydrodynamic diameter (nm)	89 ± 0.5	116 ± 0.6	115 ± 0.6	143 ± 3.8
PDI	0.36	0.39	0.23	0.29
Zeta potential (mV)	−46 ± 0.7	−37 ± 0.3	−40 ± 0.6	−33 ± 0.8

## Supplementary Materials

The following are available online at https://www.mdpi.com/2079-4991/10/6/1016/s1, Figure S1: Experimental set up of the in vitro experiments, Figure S2: Characterization of the MNP formulations by dynamic light scattering (DLS). Determination of the hydrodynamic diameter of the MNP formulations using size distribution by intensity and number, Figure S3: Temperature distribution maps (heat maps) of tumor surface temperatures of the two consecutive hyperthermia treatments. The temperature distribution of the tumor surface (in percentage) is plotted against the respective relative tumor volume at day 28 for each animal after the first (striped bars) and second hyperthermia treatment (plain colored bars) for MF66 and MF66-DOX treated BT474 xenografts, Figure S4: Internalization of MNP formulations in BT474 cancer cells. Representative fluorescence microscopic images of internalized free ligand DOX and MNP formulations by BT474 cells in vitro. Cells (each 1 × 104 cells) were incubated with different MNP formulations (MF66-DOX-N6L: 40 µmol DOX/g iron, 3.5 µmol N6L/g iron; MF66-DOX: 40 µmol DOX/g iron) and the free ligand DOX in comparable concentrations as in the functionalized modality for 24 h. Fluorescence microscopic analysis (excitation 480–485 nm; emission 580–595 nm). Red arrows: Internalized MNPs. Scale bar: 20 µm.
