# Supplementary material for: Iron Oxide Nanoparticles as Carriers for DOX and Magnetic Hyperthermia after Intratumoral Application into Breast Cancer in Mice: Impact and Future Perspectives
Source: Nanomaterials (Basel). 2020 May 26;10(6):1016. doi: 10.3390/nano10061016 (PMC7352767; doi:10.3390/nano10061016)
Supplement: Supplementary file 1 [file nanomaterials-10-01016-s001.pdf]

Supplemental Materials

Iron Oxide Nanoparticles as Carriers for DOX and Magnetic Hyperthermia after Intratumoral Application into Breast Cancer in Mice: Impact and Future Perspectives

Susann Piehler [1], Heidi Dähring [1], Julia Grandke [1], Julia Göring [1], Pierre Couleaud [2], Antonio Aires [2], Aitziber L. Cortajarena [2,3,4], José Courty [5], Alfonso Latorre [2], Álvaro Somoza [2], Ulf Teichgräber [1] and Ingrid Hilger [1,]*

[1] Institute for Diagnostic and Interventional Radiology, Jena University Hospital—Friedrich Schiller University Jena, D-07747 Jena, Germany; susann.piehler@med.uni-jena.de (S.P.); dheidi@t-online.de (H.D.); julia.grandke@gmail.com (Julia Grandke); julia.goering@med.uni-jena.de (Julia Göring); ulf.teichgraeber@med.uni-jena.de (U.T.)

[2] IMDEA Nanociencia & Nanobiotechnology Associated Unit (CNB-CSIC-IMDEA), 28049 Madrid, Spain couleaud.pierre@gmail.com (P.C.); antonio.aires@imdea.org (A.A.); aitziber.lopezcortajarena@imdea.org (A.L.C.); alvaro.somoza@imdea.org (A.S.); alfonso.latorre@imdea.org (A.L.)

[3] Center for Cooperative Research in Biomaterials (CIC biomaGUNE), Parque Tecnológico de San Sebastián, 20014 Donostia-San Sebastián, Spain

[4] Ikerbasque, Basque Foundation for Science, 48013 Bilbao, Spain

[5] Laboratoire Croissance, Réparation et Régénération Tissulaire (CRRET), Université Paris EST Créteil, 94010 Créteil, France; courty@u-pec.fr

* Correspondence: ingrid.hilger@med.uni-jena.de; Tel.: 0049-3641-9325921

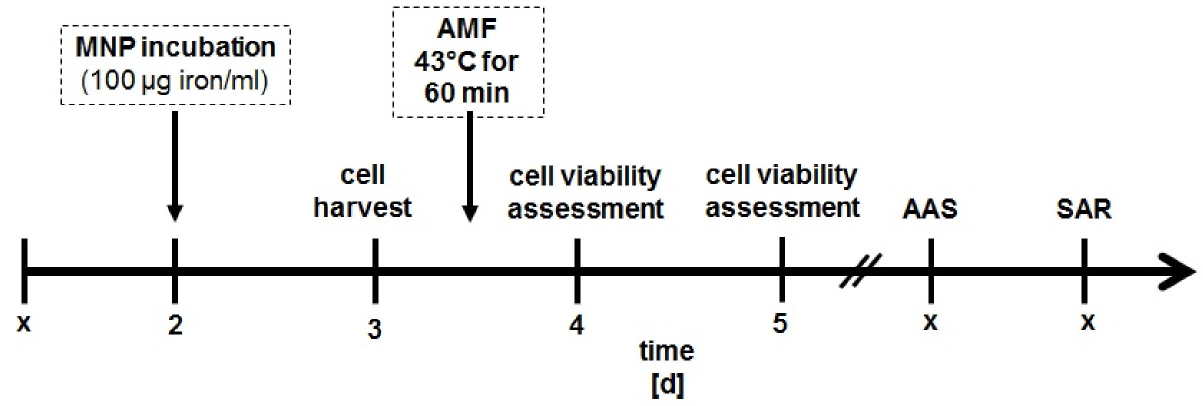

Figure S1. Experimental set up of the *in vitro* experiments.

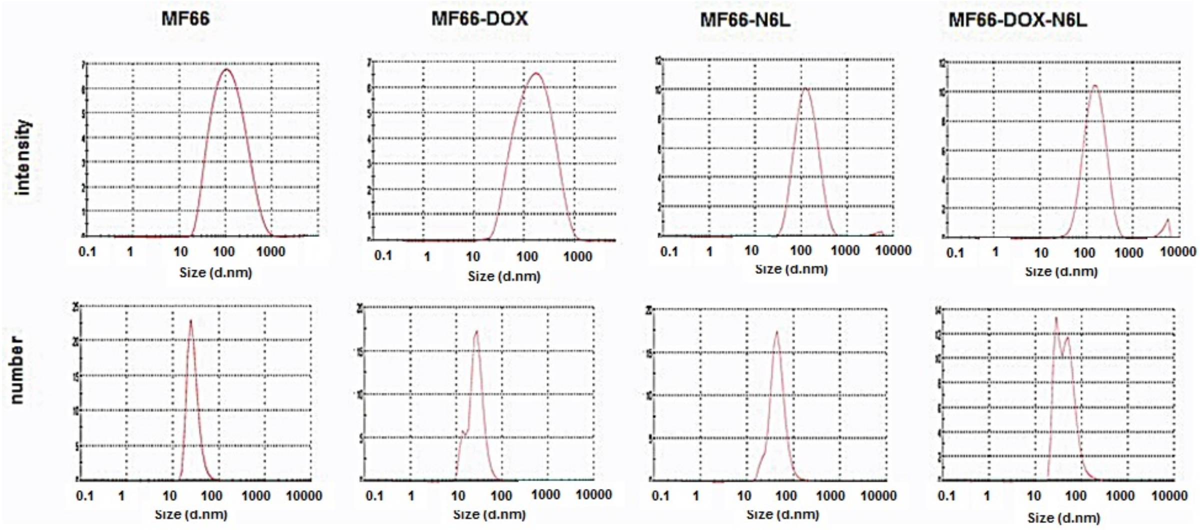

Figure S2. Characterization of the MNP formulations by dynamic light scattering (DLS). Determination of the hydrodynamic diameter of the MNP formulations using size distribution by intensity and number.

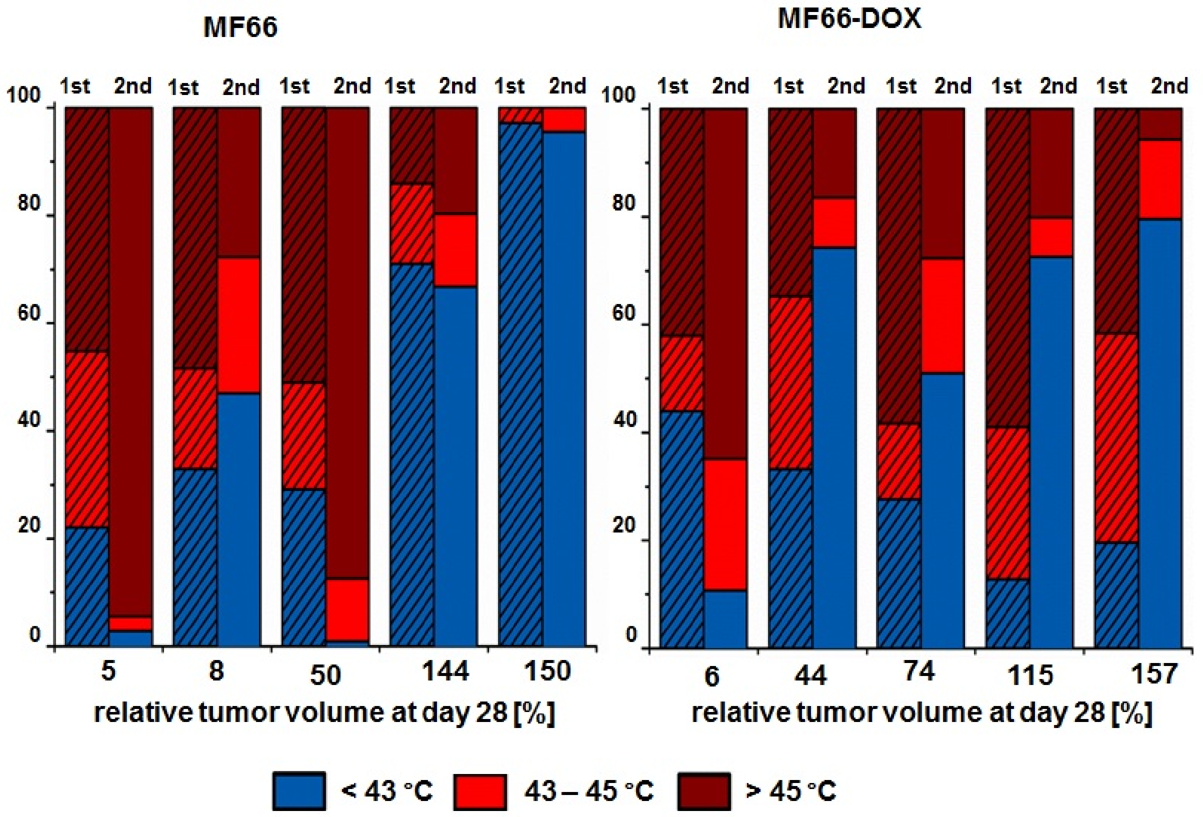

Figure S3. Temperature distribution maps (heat maps) of tumor surface temperatures of the two consecutive hyperthermia treatments. The temperature distribution of the tumor surface (in percentage) is plotted against the respective relative tumor volume at day 28 for each animal after the first (striped bars) and second hyperthermia treatment (plain colored bars) for MF66 and MF66-DOX treated BT474 xenografts.

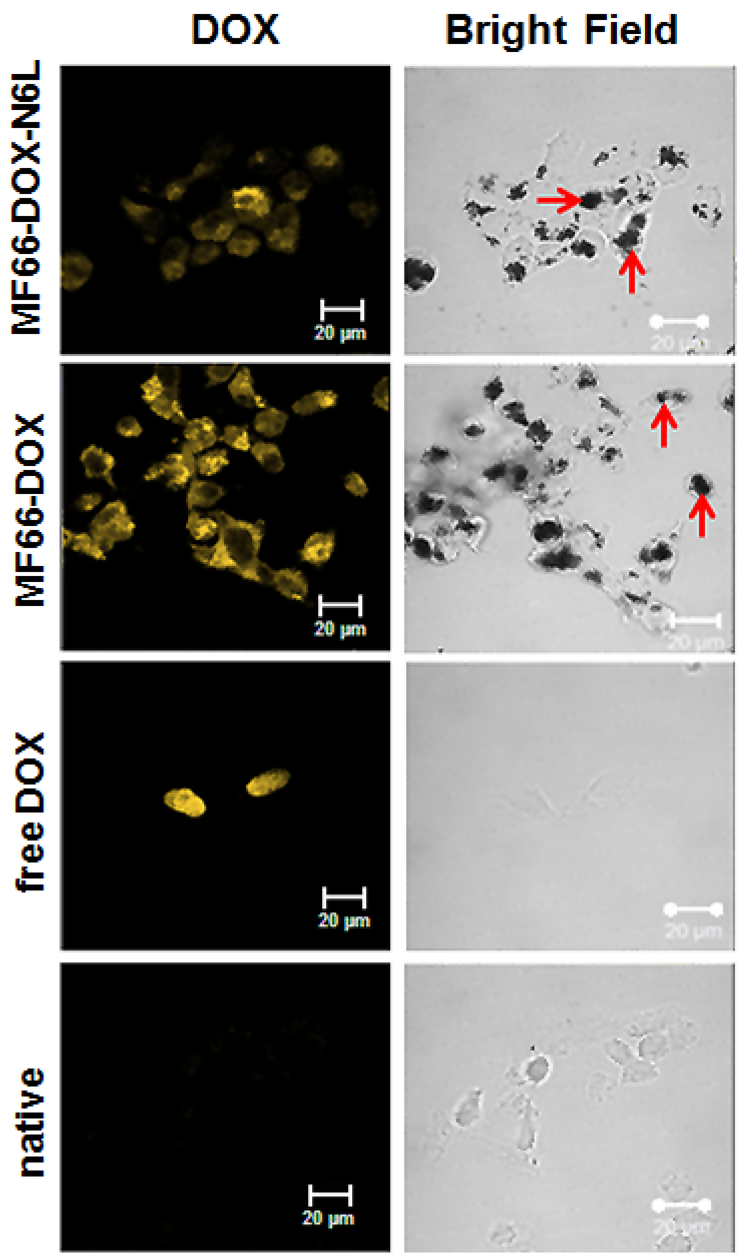

Figure S4. Internalization of MNP formulations in BT474 cancer cells. Representative fluorescence microscopic images of internalized free ligand DOX and MNP formulations by BT474 cells *in vitro*. Cells (each 1 × 10^4 cells) were incubated with different MNP formulations (MF66-DOX-N6L: 40 µmol DOX/g iron, 3.5 µmol N6L/g iron; MF66-DOX: 40 µmol DOX/g iron) and the free ligand DOX in comparable concentrations as in the functionalized modality for 24 h. Fluorescence microscopic analysis (excitation 480–485 nm; emission 580–595 nm). Red arrows: Internalized MNPs. Scale bar: 20 µm.
